# Number Word Use in Toddlerhood Is Associated with Number Recall Performance at Seven Years of Age

**DOI:** 10.1371/journal.pone.0098573

**Published:** 2014-06-03

**Authors:** Melissa E. Libertus, Peter B. Marschik, Christa Einspieler

**Affiliations:** 1 Department of Psychology, Learning Research and Development Center, University of Pittsburgh, Pittsburgh, Pennsylvania, United States of America; 2 Institute of Physiology, Developmental Physiology and Developmental Neuroscience, Center for Physiological Medicine, Medical University of Graz, Graz, Austria; University Children's Hospital Tuebingen, Germany

## Abstract

Previous studies have shown that verbal working memory and vocabulary acquisition are linked in early childhood. However, it is unclear whether acquisition of a narrow range of words during toddlerhood may be particularly related to recall of the same words later in life. Here we asked whether vocabulary acquisition of number words, location and quantifier terms over the first three years of life are associated with verbal and visuospatial working memory at seven years. Our results demonstrate that children who produced more number words between 20–26 months and started to produce the number words 1–10 earlier showed greater number recall at 7 years of age. This link was specific to numbers and neither extended to quantifier and location terms nor verbal and visuospatial working memory performance with other stimuli. These findings suggest a category-specific link between the mental lexicon of number words and working memory for numbers at an early age.

## Introduction

Numerous neuropsychological and developmental studies have shown that verbal working memory and vocabulary acquisition are linked; for a review, see [1]. For example, patient PV who was diagnosed with a pure verbal working memory deficit failed to learn word pairs between her native and a new language although her ability to learn word pairs in her native language were preserved [2]. Moreover, Gathercole and Baddeley [3] showed that children's verbal working memory at four years of age predicted their vocabulary scores one year later even when controlling for vocabulary scores at the time of the working memory assessment. Furthermore, these authors also showed that children with low verbal working memory capacity were slower to learn and had worse retention rates for phonologically unfamiliar names [4]. Similarly, Michas and Henry [5] showed that verbal working memory was a significant predictor of 5-year-old children's ability to produce and comprehend new, explicitly taught words one week later. However, this relationship seems to reverse after five years of age when vocabulary scores are a better predictor of later verbal working memory performance than the converse [6], [7]. These findings suggest that verbal working memory may be especially important for early vocabulary development for instance by facilitating the establishment of long-term memory representations of new words [3]. With age, however, other processes such as semantic and conceptual skills may become more important for vocabulary acquisition than verbal working memory [6]. The only exception to this rule seems to be the case of second language acquisition where verbal working memory and vocabulary acquisition remain linked throughout childhood and into adulthood [8]–[10].

While these previous findings suggest the importance of vocabulary knowledge for verbal working memory performance after the age of five years, it is unclear whether early acquisition of specific words leads to better recall of the same words in a memory task later in life. All of the previous studies have taken a general approach to assessing the relationship between verbal working memory and vocabulary acquisition by either using non-word repetition, non-word memory span, or other traditional working memory span tasks such as digit or letter span tasks to assess verbal working memory and broad measures of vocabulary such as the standardized British Picture Vocabulary Scale [11]. Furthermore, none of these studies have looked at vocabulary knowledge prior to four years of age. Thus, it is still an open question whether early acquisition of a narrow range of words during toddlerhood may be particularly related to recall of the same words in a verbal working memory task later in life. One clearly demarcated category of words that lends itself to answer such a question can be found in the case of number words. Children usually start to produce number words by the age of two years, even though it is often not until the age of three or older that children actually understand the meaning of these utterances [12].

In the present study we ask whether the age at which children first produce number words and the amount of number words that they acquire over the first three years of life are associated with later verbal working memory performance on a number recall task at seven years of age. Moreover, we are interested in whether other categories of early vocabulary such as spatial and quantifier terms are also related to number recall performance later in life. Finally, we ask whether early number word, spatial and quantifier term acquisition is predictive of other types of verbal and visuospatial working memory than number recall.

To this end, we asked parents to complete the Austrian Communicative Development Inventory (ACDI-2) [13], [14] every two months while their children were between 20 and 36 months of age. The ACDI-2 measures production of number words, quantifier and location terms amongst other lexical and grammatical items as well as communicative strategies. Subsequently, children's verbal and visuospatial working memory was assessed on four different tasks at the age of seven years. We hypothesized that if there were a general link between vocabulary knowledge and verbal working memory, we would find that vocabulary acquisition of all categories in toddlerhood would be linked to verbal working memory performance with different stimuli later in life. However, if for example number word acquisition in toddlerhood were only linked to verbal working memory for numbers later in life, this would suggest a category-specific link between this particular class of words and its importance for recall from working memory later in life.

## Method

### Participants

Twenty-five children (16 females) and at least one of their parents who were enrolled in the comprehensive Developmental Physiology and Developmental Neuroscience (DPDN) longitudinal study that examines various aspects of motor, cognitive, and language development provided data for the present study. The study was reviewed and approved by the Ethics Committee of the Medical University of Graz. The DPDN originally included 62 children who were all recruited as neonates born at term within a 3-week-interval (end of August to middle of September 1998) in Graz (University Hospital and three maternity units). Only 36 children participated from 12 months onwards and by age 7 years only 25 children remained.

Five parents (20.0%) had earned a college degree, fourteen parents (56.0%) had an advanced high school degree required for university admissions (“Gymnasialabschluss”), and five parents (20.0%) had a high school degree. Educational information from one parent was missing. Parents of all children provided informed written consent prior to their child's participation and children provided verbal assent before testing at the age of seven years. All children received a small gift (e.g., small toy or book) to thank them for their participation.

### Materials

#### Austrian Communicative Development Inventory, second edition (ACDI-2)

The ACDI-2 [13], [14] is an adaptation of the MacArthur-Bates Communicative Development Inventories [15] for Austrian-German. It is a parental questionnaire designed to assess expressive language acquisition and communication skills in children between the ages of 18 months and 3 years and is comprised of two different parts. Part 1 consists of word lists of 23 different semantic or grammatical categories where parents check words that they have heard their children say. Part 2 asks questions about morphological and syntactical capacities. For the purposes of the present study, only scores on the wordlist subscales for prepositions and locations (section 19, 35 items), quantifiers and determiners (section 20, 18 items), and number words (section 23, 20 items) were included (see File_S1). Independent studies demonstrate that parental reports of children's language development are reliable [16]–[20].

#### Bayley Scales of Infant and Toddler Development, second edition (BSID-II)

The BSID-II [21] was administered to assess children's mental development at age 24 months to control for general cognitive development in toddlerhood. It is standardized for children between 1 and 42 months of age and provides individual scores on a mental development index (MDI) and a psychomotor development index (PDI). Each index is standardized to a mean of 100 and a standard deviation of 15. Only the MDI was used in the present study.

#### Kaufman Assessment Battery for Children, German Version

The K-ABC German adaptation [22] was administered to assess children's cognitive development at seven years of age. It contains subscales for sequential and simultaneous processing as well as planning, learning, and knowledge. For the purposes of the present study, we only focused on raw scores on two verbal working memory tasks – the number recall task and the word order task – and two visuospatial working memory tasks – the spatial memory task and the hand movements task. In the number recall task, a trained experimenter verbally presents the child with between two and nine digits and the child is asked to verbally recall them in the correct order. In the word order task, the child is verbally presented with a sequence of two to four common object words and is asked to subsequently touch images of these objects in the correct order in a set of five to seven images. In the spatial memory task, children are shown a grid with two to seven objects and they are subsequently asked to indicate the locations of the objects on an empty grid of 3×3 or 3×4 cells. The identity of the objects is irrelevant to this task. In the hand movements task, the child is asked to copy a series of taps that the experimenter makes with the fist, the palm, or the side of the hand. There are a total of 20 items for each task, and administration is discontinued after a child has failed all items of a given complexity.

### Procedure

Parents completed the ACDI-2 every two months when their child was between 20 and 36 months of age, yielding a total of nine possible ACDI-2 data sets for each child. Questionnaires were mailed home to the parents and had to be returned within three days. Additionally, children were tested by a trained experimenter on the BSID-II at the age of 24 months (mean age  =  24 months, SD  =  14 days) and on the K-ABC at 7 years of age (mean age  =  7.02, SD  =  0.01).

### Data analysis

For the three different ACDI-2 subscales of interest (number words, location and quantifier terms), we calculated the average number of words that parents reported their child to produce in each subscale at each of the nine time points between 20–36 months of age. We also calculated the average age of onset of number words, location and quantifier terms based on the earliest age at which parents reported the production of the words included in each subscale. Parents of five children did not report any production of number words on any of the ACDI-2s. Furthermore, only six parents reported any production of the number words 11-15 and only three parents reported production of any of the number words 16–20 on any of the ACDI-2s yielding too few data points for these number words to be included for further analyses. Thus, we only calculated the average age of number word onset for children whose parents reported production of any number words between 1–10 on any of the ACDI-2s.

We used the standardized BSID-II mental index as a control measure of children's mental development in toddlerhood. BSID-II scores from six children were unavailable due to missing the 24-month visit (n = 5) or non-compliance during the assessment (n = 1).

We used the raw scores from the K-ABC number recall and word order tasks as measures of verbal working memory, and the raw scores from the K-ABC spatial memory and hand movements tasks as measures of visuospatial working memory at seven years of age. K-ABC scores from these four tasks were unavailable for four children due to missing the 7-year visit (n = 3) or non-compliance during the assessment (n = 1). All data is available from the authors upon request.

## Results

### Descriptive statistics

The average number of words that parents reported their child to produce in the three Austrian Communicative Development Inventory (ACDI-2) [13], [14] subscales of interest (number words, quantifier and location terms) at each of the nine time points can be found in Table 1. The average age of onset for the number words 1–10 was 25.54 months (SD  =  4.08 months), for quantifier terms it was 26.85 months (SD  =  2.70 months), and for location terms it was 26.77 months (SD  =  3.32 months). At age 24 months, children had an average mental development index of 121.00 (SD  =  11.62) on the Bayley Scales of Infant and Toddler Development (BSID-II) [21].

**Table 1:** ACDI-2 subscale scores by age.

Age	Number words (max. score 20)	Location terms (max. score 35)	Quantifier terms (max. score 18)
20 months (n = 23)	1.26 (2.73)	5.48 (4.20)	1.70 (2.01)
22 months (n = 23)	2.70 (4.32)	9.43 (7.51)	4.35 (3.52)
24 months (n = 24)	3.96 (4.51)	13.46 (8.94)	6.17 (4.71)
26 months (n = 23)	4.87 (4.63)	16.95 (9.91)	7.95 (4.57)
28 months (n = 24)	6.63 (5.49)	19.91 (10.60)	9.91 (4.76)
30 months (n = 25)	7.00 (6.04)	23.22 (10.58)	11.86 (4.46)
32 months (n = 23)	7.39 (6.26)	26.68 (8.13)	13.65 (2.94)
34 months (n = 24)	7.79 (6.18)	28.54 (7.24)	13.86 (3.30)
36 months (n = 22)	9.05 (6.58)	31.87 (4.37)	15.62 (2.01)

Average number of words and standard deviations in parentheses that parents reported their child to use in the three ACDI-2 subscales of interest (number words, location and quantifier terms) at each of the nine time points.

At age seven years, children scored on average 11.0 points (SD  =  3.05) on the number recall task of the Kaufman Assessment Battery for Children, German Version (K-ABC) [22] and 13.76 points (SD  =  3.42) on the word order task of the K-ABC. They scored on average 13.71 points (SD  =  1.45) on the spatial memory task of the K-ABC and 13.29 points (SD  =  2.13) on the hand movements task of the K-ABC.

### Correlations between average number of words in toddlerhood and memory at 7 years

#### Number words

The average amount of number words that parents reported their child to produce at 20, 22, 24, and 26 months of age was positively correlated with number recall at 7 years (20 months: r(17)  =  .87, *p*<.001; 22 months: r(17)  =  .71, *p* = .001; 24 months: r(18)  =  .50, *p*<.05; 26 months: r(17)  =  .70, *p* = 0.001), i.e., the more number words the child used between 20–26 months of age, the greater the child's ability to recall numbers at 7 years of age. These results remain statistically significant using FDR-correction for multiple comparisons, except at 24 months where it is only marginally significant (*p* = .056). The correlations at 20, 22, and 26 months remained significant even when controlling for the mental development index on the BSID-II at 24 months of age, parental education, and age at the time of K-ABC administration (all r_par_s(10) > .57, *p*s < .05). The correlation at 24 months remained marginally significant when controlling for the mental development index on the BSID-II at 24 months of age, parental education, and age at the time of K-ABC administration (r_par_(10)  =  .50, *p*<.10). The average amount of number words that parents reported their child to use at 28, 30, 32, 34, and 36 months was not correlated with number recall at 7 years (28 months: r(18)  =  .39, *p* = .09; 30 months: r(19)  =  .36, p = .11; 32 months: r(18)  =  .26, *p* = .27; 34 months: r(18)  =  .27, *p* = .26; 36 months: r(16)  =  .25, *p* = .31).

The only other memory scores at seven years that were correlated with number word scores at any point during toddlerhood even when controlling for the mental development index on the BSID-II at 24 months of age, parental education, and age at the time of K-ABC administration were the hand movements and word order scores and the number word scores at 20 months (hand movements: r_par_(10)  =  .93, *p*<.001; word order: r_par_(10)  =  .72, *p*<.01).

#### Location terms

The average number of location terms that parents reported their child to produce at any age between 20–36 months did not correlate with any of the memory scores at seven years of age.

#### Quantifier terms

The average number of quantifier terms that parents reported their child to produce at 32 and 36 months of age were negatively correlated with their spatial memory scores at 7 years (32 months: r(15)  =  −.50, *p*<.05; 36 months: r(15)  =  −.55, *p*<.05), i.e., the more quantifier terms children produced at 32 and 36 months of age, the lower their spatial memory scores at 7 years. These results are no longer statistically significant when correcting for multiple comparisons using the FDR-correction method. The correlation at 36 months remained significant even when controlling for the mental development index on the BSID-II at 24 months of age, parental education, and age at the time of K-ABC administration (r_par_(10)  =  −.58, *p*<.05), but the correlation at 32 months was only marginally significant when controlling for these additional factors (r_par_(10)  =  −.57, *p* = .07). None of the other memory scores at seven years were correlated with the number of quantifier terms that parents reported their child to produce at any of the time points during toddlerhood.

### Correlations between onset of word production in toddlerhood and memory at seven years

The average age of onset of number words 1-10 was significantly correlated with the average age of onset of quantifier terms (r(18)  =  .47, *p*<.05) and the average age of onset of location terms (r(18)  =  .57, *p*<.01) respectively. The average age of onset of quantifier terms was also positively correlated with the average age of onset of location terms (r(18)  =  .76, *p*<.001).

#### Number words

**Figure 1:**
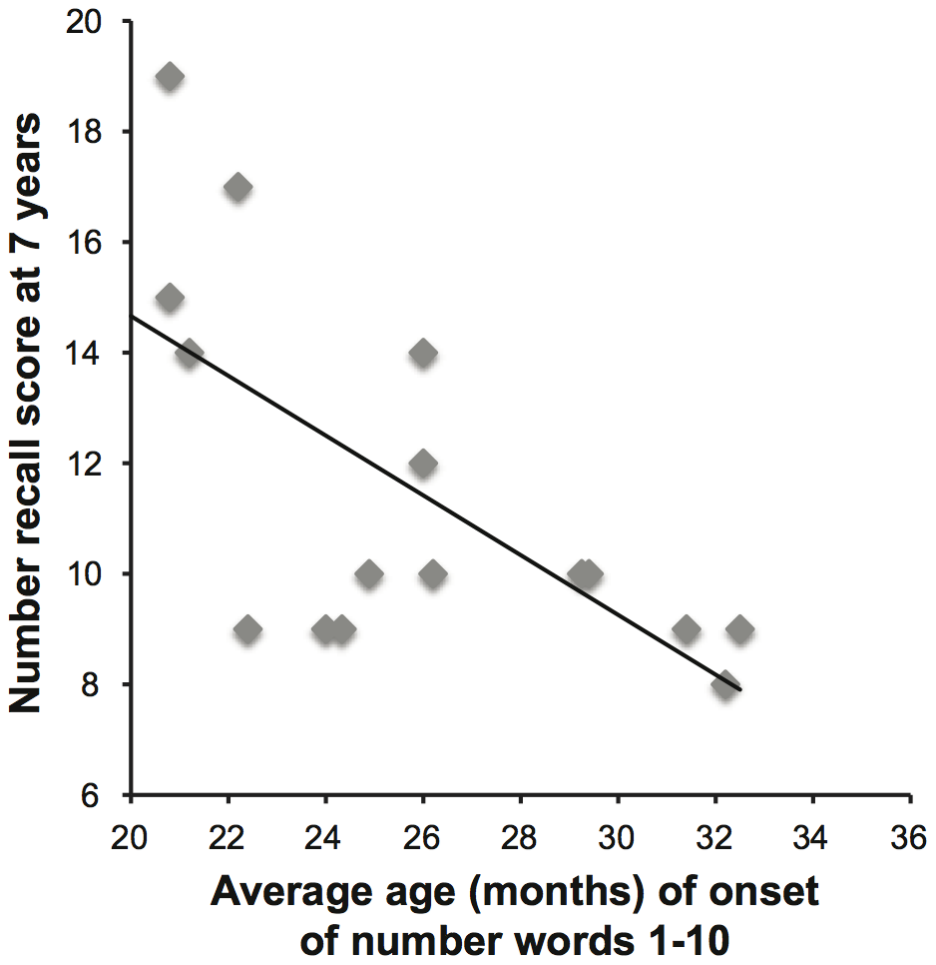
Relation between onset of number word use and later number recall. Scatterplot depicting the average age of onset of the number words 1–10 over the first three years of life and number recall scores at seven years of age.

As can be seen in Figure 1, the average age of onset of number words 1–10 was negatively correlated with number recall at seven years (r(14)  =  −.66, *p*<.01), i.e., children who started to produce the number words 1–10 at a younger age before age three years showed greater number recall than children who started to produce the number words 1–10 later during the first three years of life. This correlation remained significant even when controlling for the mental development index on the BSID-II at 24 months of age, parental education, and age at the time of K-ABC administration (r_par_(10)  =  −.60, *p*<.05).

In contrast, word order recall and hand movements scores at seven years of age were correlated with the average age of onset of number words 1–10 (word order recall: r(14)  =  −.58, *p*<.05; hand movements: r(14)  =  −.56, *p*<.05), but these correlations were no longer significant when controlling for the mental development index on the BSID-II at 24 months of age, parental education, and age at the time of K-ABC administration (word order recall: r_par_(10)  =  −.50, *p* = .10; hand movements: r_par_(10)  =  −.44, *p* = .15). Spatial memory scores at seven years of age were not correlated with the average age of onset of number words 1–10 at all (r(14)  =  −.001, *p* = .997). The results of the zero-order correlations remained unchanged when correcting for multiple comparisons using the FDR-correction method.

A direct comparison using Fisher's r-to-z transformation showed a significant difference in the zero-order correlation coefficients for average age of onset of number words with number recall and spatial memory scores respectively (Z = −2.31, *p*<.05) but not with word order recall (Z = −0.57, *p* = .57) or hand movements (Z = −0.66, *p* = .51). Moreover, the same pattern holds when comparing differences between the partial correlation coefficients of average age of onset of number words with number recall and the three other measures (controlling for the mental development index on the BSID-II, parental education, and age at the time of K-ABC administration): spatial memory: Z = −2.23, *p*<.05; word order recall: Z = −0.58, *p* = .56; hand movements: Z = −0.93, *p* = .35.

#### Location terms

The average onset of location terms was positively correlated with spatial memory scores at seven years of age (r(19)  =  .44, *p*<.05), i.e., children who started to produce location terms at an older age showed greater spatial recall than children who started to produce these terms earlier during the first three years of life. The zero-order correlation was no longer significant when correcting for multiple comparisons using the FDR-correction method. This correlation remained significant even when controlling for the mental development index on the BSID-II at 24 months of age, parental education, and age at the time of K-ABC administration (r_par_(10)  =  .63, *p*<.05).

In contrast, number recall, word order recall, and hand movements scores at seven years of age were not correlated with the average age of onset of location terms (all rs between −.34 and .02, *p*s > .13). A direct comparison using Fisher's r-to-z transformation showed a significant difference in the zero-order correlation coefficients for average age of onset of location terms with spatial recall and number recall respectively (Z = 2.07, *p*<.05) as well as with spatial recall and word order recall (Z = 3.22, *p*<.01) but only marginally significant differences with spatial recall and hand movements (Z = 1.69, *p* = .09). A similar pattern emerges when comparing differences between the partial correlation coefficients of average age of location terms with spatial recall and the three other measures (controlling for the mental development index on the BSID-II, parental education, and age at the time of K-ABC administration): number recall: Z = 2.17, *p*<.05; word order recall: Z = 3.87, *p*<.001; hand movements: Z = −0.30, *p* = .77.

#### Quantifier terms

The average age of onset of quantifier terms was negatively correlated with word order scores at seven years of age (r(19)  =  −.45, *p*<.05), but this correlation was no longer significant when controlling for the mental development index on the BSID-II at 24 months of age, parental education, and age at the time of K-ABC administration (r(10)  =  −.17, *p* = .60). Number recall, word order recall, and hand movements scores at seven years of age were not correlated with the average age of onset of quantifier terms (all rs between −.27 and .24, *p*s > .24).

## Discussion

We found that the more number words the child produced between 20–26 months of age and the earlier the child started to produce the number words 1–10, the greater the child's ability to recall numbers at 7 years of age even when controlling for general cognitive development during toddlerhood, parental education, and age at the time of the working memory assessment. No such relationship was found between location and quantifier term production in toddlerhood and number recall later in life. Importantly, we also did not find a link between early number word production and later verbal working memory performance for other types of words. These findings provide preliminary evidence of a category-specific link between early vocabulary knowledge and later verbal working memory performance using number words as stimuli. Follow-up studies with larger sample sizes should be conducted to corroborate the present findings.

Our findings thus extend previous findings that have demonstrated a link between early verbal working memory abilities for vocabulary acquisition before the age of five years in two important ways [3], [6], [7]. First, we show that early vocabulary before age three is associated with later verbal working memory and second, that this link is restricted to specific categories of early vocabulary and later working memory tasks. It is possible that earlier production of number words leads to faster access of these words in long-term memory later in life, which in turn may free additional processing resources during the number recall task. This view is supported by the fact that picture-naming latency is predicted by the age at which the word was learned [23]. Another possible explanation is that children who start to produce number words earlier in life also acquire the meaning of these words earlier and are able to develop more advanced strategies such as chunking to aid recall of numbers later in life. More work including a larger and more varied sample is needed to understand why the production of number words 1–10 after 26 months of age was not predictive of later number recall and whether this link holds for number words beyond this range. Interestingly, even though quantifiers are used in a very similar syntactic and semantic context to number words [24], there is no evidence that production of quantifier terms in toddlerhood is linked to number recall at seven years of age.

Surprisingly, we found a different relationship between early word production and later visuospatial working memory abilities. We found that children who started to produce location terms at an older age showed greater spatial recall than children who started to produce these terms earlier during the first three years of life, and that the more quantifier terms children produced at 36 months of age, the lower their spatial memory scores at seven years even when controlling for general cognitive development during toddlerhood, parental education, and age at the time of the working memory assessment. These findings suggest that early production of location terms and the number of quantifier terms that children produce may actually inhibit spatial memory abilities. One possible explanation could be that children who acquired location and quantifier terms earlier try to use more verbally mediated strategies to solve the spatial working memory task at seven years, which in turn may lead to decreased success. This view is supported by the fact that children at seven years of age and older are more likely to use verbal recoding strategies to recall visual patterns [25], [26]. However, more work is needed to assess the reliability of our unexpected findings also given that they did not survive a correction for multiple comparisons.

In sum, we found an intriguing link between early number word production in toddlerhood and later verbal working memory for numbers at seven years of age. Children who produced more number words between 20–26 months of age and started to produce the number words 1–10 earlier showed a greater ability to recall numbers at 7 years of age even when controlling for general cognitive development during toddlerhood, parental education, and age at the time of the working memory assessment. This link was specific to number words and number recall and neither extends to quantifier and location terms nor verbal and visuospatial working memory performance with other types of stimuli. In conjunction with previous findings demonstrating a link between early verbal working memory and later vocabulary acquisition [3], our findings suggest a category-specific link between vocabulary and verbal working memory at a very young age.

## Supporting Information

File S1 **Austrian Communicative Development Inventory 2: Items for subscales 2.19 (Prepositions and location terms), 2.20 (Quantifiers and determiners), and 2.23 (Number words).**
(DOCX)

## References

[1] Baddeley A (2003) Working memory and language: an overview. Journal of Communication Disorders 36: 189–208. 12742667 10.1016/s0021-9924(03)00019-4

[2] Baddeley A, Papagno C, Vallar G (1988) When long-term learning depends on short-term storage. Journal of Memory and Language 27: 586–595.

[3] Gathercole SE, Baddeley A (1989) Evaluation of the role of phonological STM in the development of vocabulary in children: A longitudinal study. Journal of Memory and Language 28: 200–213.

[4] Gathercole SE, Baddeley A (1990) The role of phonological memory in vocabulary acquisition: A study of young children learning new names. British Journal of Psychology 81: 439–454.

[5] Michas IC, Henry LA (1994) The link between phonological memory and vocabulary acquisition. British Journal of Developmental Psychology 12.

[6] Gathercole SE, Willis CS, Emslie H, Baddeley A (1992) Phonological Memory and Vocabulary Development During the Early School Years: A Longitudinal Study. Developmental Psychology 28: 887–898.

[7] Jarrold C, Baddeley A, Hewes AK, Leeke TC, Phillips CE (2004) What links verbal short-term memory performance and vocabulary level? Evidence of changing relationships among individuals with learning disability. Journal of Memory and Language 50.

[8] Papagno C, Valentine T, Baddeley A (1991) Phonological short-term memory and foreign-language vocabulary learning. Journal of Memory and Language 30: 331–347.

[9] Atkins PWB, Baddeley A (1998) Working memory and distributed vocabulary learning. Applied Psycholinguistics 19: 537–552.

[10] Gathercole SE, Service E, Hitch GJ, Adams A-M, Martin AJ (1999) Phonological short-term memory and vocabulary development: further evidence on the nature of the relationship. Applied Cognitive Psychology 13: 65–77.

[11] Dunn LM, Dunn LM (1982) British Picture Vocabulary Scale. Windsor, England: nfer Nelson.

[12] Fuson KC (1988) Children's counting and concepts of number; Brainerd CJ, editor. New York: Springer-Verlag.

[13] Marschik PB, Einspieler C, Garzarolli B, Prechtl HFR (2007) Events at early development: are they associated with early word production and neurodevelopmental abilities at the preschool age? Early Human Development 83: 107–114. 16876340 10.1016/j.earlhumdev.2006.05.009

[14] Marschik PB, Vollmann R, Einspieler C (2004) Austrian Communicative Development Inventories, Level 2. Medizinische Universität Graz.

[15] Fenson L, Dale PS, Reznick JS, Bates E, Thal DJ, et al (1994) Variability in early communicative development. Monographs of the Society for Research in Child Development 59: 1–173. 7845413

[16] Reznick JS, Goldsmith L (1989) A multiple form word production checklist for assessing early language. Journal of Child Language 16: 91–100. 2925817 10.1017/s0305000900013453

[17] Dale PS, Bates E, Reznick JS, Morisset C (1989) The validity of a parent report instrument of child language at twenty months. Journal of Child Language 16: 239–249. 2760125 10.1017/s0305000900010394

[18] Ring ED, Fenson L (2000) The correspondence between parent report and child performance for receptive and expressive vocabulary beyond infancy. First Language 20: 141–159.

[19] Rescorla L, Alley A (2001) Validation of the language development survey (LDS): a parent report tool for identifying language delay in toddlers. Journal of Speech, Language and Hearing Research 44: 434–445. 10.1044/1092-4388(2001/035) 11324663

[20] Libertus ME, Odic D, Feigenson L, Halberda J (in press) A Developmental Vocabulary Assessment for Parents (DVAP): Validating parental report of vocabulary size in 2- to 7-year-olds. Journal of Cognition and Development.

[21] Bayley N (1993) Bayley Scales of Infant and Toddler Development, 2nd edition. San Antonio, TX: Pearson.

[22] Melchers P, Preuβ U (2003) K-ABC Kaufman assessment battery for children. German version. Leiden: PITS.

[23] Carroll JB, White MN (1973) Word frequency and age of acquisition as determiners of picture-naming latency. Quarterly Journal of Experimental Psychology 25: 85–95.

[24] Wynn K (1992) Children's Acquisition of the Number Words and the Counting System. Cognitive Psychology 24: 220–251.

[25] Miles C, Morgan MJ, Milne AB, Morris EDM (1996) Developmental and Individual differences in Visual Memory Span. Current Psychology 15: 53–67.

[26] Pickering SJ, Gathercole SE, Hall M, Lloyd SA (2001) Development of memory for pattern and path: Further evidence for the fractionation of visuo-spatial memory. Quarterly Journal of Experimental Psych ology 54: 397–420. 10.1080/713755973 11394054

