# Supplementary material for: Number Word Use in Toddlerhood Is Associated with Number Recall Performance at Seven Years of Age
Source: PLoS One. 2014 Jun 3;9(6):e98573. doi: 10.1371/journal.pone.0098573 (PMC4044038; doi:10.1371/journal.pone.0098573)
Supplement: File S1 — Austrian Communicative Development Inventory 2: Items for subscales 2.19 (Prepositions and location terms), 2.20 (Quantifiers and determiners), and 2.23 (Number words). (DOCX) [file pone.0098573.s001.docx]

**Supporting Information**

**“Number word use in toddlerhood is associated with number recall performance at seven years of age”**

Melissa E. Libertus, Peter B. Marschik, & Christa Einspieler

**Austrian Communicative Development Inventory 2 – Subscale items**

2.19: Prepositions and location terms

1. ab (from)
2. an (by)
3. auf (on)
4. aus (out of)
5. bei (next to / near)
6. bis (by, until)
7. da (there)
8. durch (through)
9. für (for)
10. (he)rauf (up)
11. (he)raus (out)
12. (he)rein (into)
13. (he)runter (down)
14. hier (here)
15. hinauf (up)
16. hinein (in / inside)
17. hinter, hinten (behind)
18. hinunter (down)
19. in (in)
20. mit (with)
21. nach (after)
22. neben (beside)
23. oben (on top of)
24. seit (for, since)
25. über (above)
26. um (about)
27. um … herum (around)
28. unter (under)
29. von (of)
30. vor (in front of)
31. während (during)
32. weg (away)
33. wegen (concerning)
34. zu (to / toward)
35. zurück (back)

2.20: Quantifiers and determiners

1. alle (all)
2. auch (too)
3. der (the – masculine)
4. die (the – female)
5. das (the – neuter)
6. ein (a / an)
7. ein bißchen (a bit)
8. ein paar (a few)
9. ein wenig (a little)
10. einige (some)
11. etliche (several)
12. etwas (any)
13. jeder (each / every)
14. kein (none)
15. mehr (more)
16. nichts (nothing)
17. viel (a lot)
18. viele (much)

2.23: Number words

1. 1
2. 2
3. 3
4. 4
5. 5
6. 6
7. 7
8. 8
9. 9
10. 10
11. 11
12. 12
13. 13
14. 14
15. 15
16. 16
17. 17
18. 18
19. 19
20. 20
